# The Impact of the *Fusarium* Mycotoxin Deoxynivalenol on the Health and Performance of Broiler Chickens

**DOI:** 10.3390/ijms12117996

**Published:** 2011-11-16

**Authors:** Wageha A. Awad, Michael Hess, Magdalena Twarużek, Jan Grajewski, Robert Kosicki, Josef Böhm, Jürgen Zentek

**Affiliations:** 1 Department of Veterinary Medicine, Institute of Animal Nutrition, Freie Universität Berlin, 14195 Berlin, Germany; E-Mail: Zentek.Juergen@vetmed.fu-berlin.de; 2 Clinic for Avian, Reptile and Fish Medicine, Department for Farm Animals and Veterinary Public Health, University of Veterinary Medicine, A-1210 Vienna, Austria; E-Mail: Michael.Hess@vetmeduni.ac.at; 3 Division of Physiology and Toxicology, Institute of Experimental Biology, Kazimierz Wielki University, 85-064 Bydgoszcz, Poland; E-Mails: twarmag@ukw.edu.pl (M.T.); jangra@ukw.edu.pl (J.G.); robkos@ukw.edu.pl (R.K.); 4 Department for Farm Animals and Veterinary Public Health, Institute of Animal Nutrition, University of Veterinary Medicine, A-1210 Vienna, Austria; E-Mail: Josef.Boehm@vetmeduni.ac.at

**Keywords:** broiler, *Fusarium* mycotoxin, deoxynivalenol, small intestine, morphology, metabolism

## Abstract

The aim of the present experiment was to investigate the effects of feeding grains naturally contaminated with *Fusarium* mycotoxins on morphometric indices of jejunum and to follow the passage of deoxynivalenol (DON) through subsequent segments of the digestive tract of broilers. A total of 45 1-d-old broiler chickens (Ross 308 males) were randomly allotted to three dietary treatments (15 birds/treatment): (1) control diet; (2) diet contaminated with 1 mg DON/kg feed; (3) diet contaminated with 5 mg DON/kg feed for five weeks. None of the zootechnical traits (body weight, body weight gain, feed intake, and feed conversion) responded to increased DON levels in the diet. However, DON at both dietary levels (1 mg and 5 mg DON/kg feed) significantly altered the small intestinal morphology. In the jejunum, the villi were significantly (*P* < 0.01) shorter in both DON treated groups compared with the controls. Furthermore, the dietary inclusion of DON decreased (*P* < 0.05) the villus surface area in both DON treated groups. The absolute or relative organ weights (liver, heart, proventriculus, gizzard, small intestine, spleen, pancreas, colon, cecum, bursa of Fabricius and thymus) were not altered (*P* > 0.05) in broilers fed the diet containing DON compared with controls. DON and de-epoxy-DON (DOM-1) were analyzed in serum, bile, liver, feces and digesta from consecutive segments of the digestive tract (gizzard, cecum, and rectum). Concentrations of DON and its metabolite DOM-1 in serum, bile, and liver were lower than the detection limits of the applied liquid chromatography coupled with mass spectrometry (LC-MS/MS) method. Only about 10 to 12% and 6% of the ingested DON was recovered in gizzard and feces, irrespective of the dietary DON-concentration. However, the DON recovery in the cecum as percentage of DON-intake varied between 18 to 22% and was not influenced by dietary DON-concentration. Interestingly, in the present trial, DOM-1 did not appear in the large intestine and in feces. The results indicate that deepoxydation in the present study hardly occurred in the distal segments of the digestive tract, assuming that the complete de-epoxydation occurs in the proximal small intestine where the majority of the parent toxin is absorbed. In conclusion, diets with DON contamination below levels that induce a negative impact on performance could alter small intestinal morphology in broilers. Additionally, the results confirm that the majority of the ingested DON quickly disappears through the gastrointestinal tract.

## 1. Introduction

The mycotoxin deoxynivalenol (DON), the most prevalent trichothecene mycotoxin contaminating crops in Europe and North America [1], is commonly detected in cereals and grains. DON is a public health concern, as it is resistant to milling, processing and heating and readily enters the food chain. The digestive tract is a target for DON. The gastrointestinal mucosa serves as a dynamic barrier regulating uptake of nutrients and water, while excluding potential pathogens and toxicants [2]. Following ingestion of contaminated food or feed, intestinal epithelial cells could be exposed to a high concentration of toxicants, potentially affecting intestinal functions [3].

All animal species tested have been shown to be susceptible to DON. However, the degree of susceptibility varies according to the following order: pigs > mice > rats > poultry ≈ ruminants [4]. The difference in sensitivity may be explained by differences in absorption, distribution, metabolism, and elimination of DON [5]. In general, poultry are less sensitive to DON compared to other species [6,7]. Negative effects on the performance of broilers occurred in dietary concentrations greater than 5 ppm, but in some investigations [8,9] even higher concentrations did not consistently induce clinical signs. One important aspect of DON toxicity is an injury of the gastrointestinal tract.

Although it is a food and feed contaminant, only scarce reports exist on the gastrointestinal effects of DON [10–14]. Critical gaps still exist regarding the potential effects of DON, and widespread animal exposure needs additional research to improve capacity for assessing adverse effects of DON. Understanding the effects of DON on the gastrointestinal tract is of major importance for protecting animal health and risk assessment.

Prelusky *et al*. [4] reported some changes in pigs fed a diet containing low levels of DON, including alterations in stomach and serum proteins, and suggesting specific effects of *Fusarium* toxins and particularly DON. The injury of the gastrointestinal tract, involving thickening of the mucosa of the stomach and a higher degree of folding, is one important characteristic of DON toxicity [15]. In addition, Awad *et al*. [12] found that feeding of broilers with DON artificially contaminated diets at 10 mg/kg altered the small intestinal morphology in the duodenum and jejunum.

The 12,13-epoxid group of the DON molecule is responsible for its toxicity [16]. Several microorganisms from the rumen and intestine have been shown to be capable of cleaving this group, and the resulting compound DOM-1 can be regarded as the detoxified metabolite of DON [17]. Deoxynivalenol has been completely transformed to de-epoxy DON after incubating DON with the contents from large intestine of hens [18]. However, only little is known about the metabolism of DON through the digestive tract of broilers.

Therefore, the aims of the present study were to investigate the effect of feeding a naturally DON contaminated diet at different levels (1 mg and 5 mg of DON/kg) on performance, organ weights, intestinal morphology and to follow the passage of DON through subsequent segments of the digestive tract with special consideration for the occurrence of de-epoxy-DON.

## 2. Materials and Methods

### 2.1. Birds, Housing and Diets

A total of 45 broiler 1-d-old chickens (Ross 308 males) was obtained from a commercial hatchery (Cobb Germany, Avimex GmbH) and randomly allotted to 3 dietary treatments (15 chicks/treatment) with each treatment having 5 replicates. The birds were housed in temperature-controlled batteries for the 35-day experimental period. The temperature was kept at 33 °C (from day 0 to day 3) and was gradually reduced (2–3 °C/week) until 23 °C was reached. A lighting program was applied as follow: 24 h for the first three days, 23 h for the next four days, and 18 h constant light schedule until the end of the experiment.

The basal diet was formulated based on wheat (50%), soybean meal (31.3%), maize (7.61%), soy oil (6.60%), and a premix with vitamins (1.20%), minerals, amino acids, and monocalcium phosphate (3.29%). Nutrient concentrations were formulated to meet or exceed minimum requirements for broilers according to the National Research Council [19] and Gesellschaft für Ernährungsphysiologie [20]. The control diet was prepared with non-contaminated wheat. The mycotoxin contaminated diet was prepared by replacing “uncontaminated” control wheat with DON contaminated wheat. The contaminated two diets contained 1 and 5 mg DON/kg compound feed. Presence of *Fusarium* toxins in wheat did not influence its feeding value. Chicks were fed the starter diets from d 1 to 14 and the grower diets from d 15 to 35. The average initial live weight was similar for all groups and amounted to 41.9 ± 0.46 g. Feed and water were provided *ad libitum*. Representative feed samples for each group (2 × 250 g) were analyzed for the content of dry matter, crude protein, crude ash and crude fiber [21] (Table 1). Deoxynivalenol and other trichothecenes (3-acetyl-deoxynivalenol, zearalanone, T-2 and HT-2 toxin were determined in the diets by the LC-MS/MS method of Błajet-Kosicka *et al*. [22]. During this analysis, a low contamination with *Fusarium* mycotoxins was also detected in control diet.

### 2.2. Traits

#### 2.2.1. Performance and Morphometric Indices of the Jejunum

Body weight (BW) and feed intake measurements were determined at weekly intervals. Body weight gain (BWG) was calculated as the difference between the final and initial bird weight during each of the weighing periods. Feed intake was calculated as the difference between the amount of feed supplied to the birds and the amount of feed that remained at the end of each feeding period. Feed conversion (feed: gain ratio) was calculated as the ratio between feed intake and BW gain for each period.

At the end of experiment, following weighing, birds were killed by cervical dislocation. Proventriculus, gizzard, heart, liver, pancreas, spleen, cecum, bursa of Fabricius, thymus, and colon were excised and weighed. The gastrointestinal tract was weighed after removal of the content by gentle squeezing.

The weight and length of the intestine were measured. For the small intestine, the cut was done from the start of the duodenal loop to the ileo-cecal junction. Density of the intestines was calculated as the ratio between the weight and the length of the intestine. The intestine density is considered as an indicator of the intestinal villi size of the mucosa layer [23].

At the end of the feeding trial, on d 35, tissue samples for histology were taken from jejunum close to the junction of Meckel’s diverticulum. The samples were fixed in 4% buffered formalin for 48 h. The processing consisted of serial dehydration, clearing and impregnation with wax. Tissue sections, 5 μm thick (three cross-sections from each bird), were cut by a microtome and were fixed on slides. A routine staining procedure was carried out using hematoxylin and eosin [24]. The slides were examined on an Olympus BX41 microscope (Olympus Corporation, Tokyo, Japan) fitted with a digital video camera (Olympus U-CMAD 3).

The images were analyzed using analySIS image software (Version 5) from color View soft Imaging System GmbH (Hamburg, Germany). The total of the intact well-oriented, crypt-villus units were selected in triplicate for each intestinal cross-section for each sample. The criterion for villus selection was based on the presence of an intact lamina propria. Villus height (VH) was measured from the tip of the villus to the villus-crypt junction, while crypt depth was defined as the depth of the invagination between adjacent villi. The villus width (VW) was measured at the middle of villus (the desistance between the two sides of the villus). The muscularis thickness was measured from the submucosa to the external layer of the intestine, and the villus-to-crypt ratio [25]. Apparent villus surface area (AVSA) was automatically calculated.

#### 2.2.2. DON and Its Metabolites Detection by LC-MS/MS

For liquid samples preparation, 1.0 mL serum or bile, 1.0 mL phosphate buffer pH 6.8, containing 2000 U β-glucuronidase per 1 milliliter of a sample, was added. For liver, feces, gizzard, cecum and rectum, 5.0 g of a sample was mixed with 8.0 mL phosphate buffer pH 6.8, containing 2000 U β-glucuronidase per 1.0 g sample. Thereafter, the samples were mixed and incubated overnight at room temperature. After incubation, the extraction of the samples were done by addition of 8.0 mL of acetonitrile for liquid samples and 32.0 mL of acetonitrile for digesta and liver, the whole mixture was stirred for 30 min and filtrated.

For samples cleaning-up, the extract (4.0 mL) was mixed with 0.040 mL zearalenone (40.0 ng) and passed through a Bond Elut^®^ Mycotoxin column (Varian, Harbor City, CA, USA). After clean-up 2.0 mL (2.5 mL serum and bile) of eluate were collected, mixed with 0.050 mL U-[^13^C_15_]-deoxynivalenol (127.0 ng) and evaporated to dryness at 40 °C under stream of nitrogen. Dry extract was reconstituted in 0.495 mL methanol: water (2:8 v/v) and analyzed by HPLC-MS/MS.

LC-MS/MS system consisted of 3200 QTRAP system from Applied Biosystems (Foster City, CA, USA) and a 1200 Series LC system from Agilent Technologies (Waldbronn, Germany). Chromatographic separations of mycotoxins were performed on a Phenomenex Gemini C18 column (150 mm × 4.6 mm, 5 μm). A gradient program was used with the mobile phase, combining solvent A (1% acetic acid in methanol, containing 5 mM ammonium acetate) and solvent B (1% acetic acid in water, containing 5 mM ammonium acetate) as follows: 30% A (initial), 30–90% A (5.5 min), isocratic period of 6 min at 90% A and column re-equilibration at 30% A (6.4 min). The flow rate was 0.7 mL/min while the injection volume was 20 μL. Chromatograms were integrated with the help of the Analyst 1.4.2 software. The detection limits for deoxynivalenol, 3-acetyl-deoxynivalenol, de-epoxy-DON (DOM-1), T-2, HT-2 toxin and zearalenone, were 7.0, 5.0, 7.0, 0.5, 1.7, and 0.1 ng/g, respectively. Concentrations of DON and DOM-1 in digesta and feces which were lower than the above indicated detection limits were considered with a concentration of zero in evaluating the data which implies that calculated mean values might be lower than the detection limits

### 2.3. Statistics

For analysis of the data, SPSS for Windows, Version 17.0 was used. A one-sample Kolmogorov-Smirnov test was used to examine the normal distribution of data. The analysis of variance (ANOVA) of the General Linear Model procedure was used for the differences in performance, intestinal histology and DON distribution by using one-way ANOVA and, subsequently, Duncan’s multiple range test. The pen with the group of 3 broilers was the experimental unit for performance data. The nature of the response exhibited by different parameters was determined by employing a polynomial contrast between mycotoxin-contaminated diet and control diet by using the regression analysis. Statements of statistical significance were based on *P* ≤ 0.05.

## 3. Results and Discussion

The contamination of cereal grains with toxic secondary metabolites of fungi, mycotoxins, is a permanent challenge in animal nutrition as health and performance of the animals may be compromised as well as the quality of animal derived food. Among the *Fusarium* mycotoxins, DON, is of special importance as it is formed at the field prior to harvest and because its occurrence cannot completely be avoided due to the major impact of weather conditions. From a practical viewpoint DON is of outstanding importance among these contaminants because of its frequent occurrence at levels high enough to cause adverse effects in animals [26,27].

The current study demonstrated the effects of feeding grains naturally contaminated with the *Fusarium* toxin deoxynivalenol on performance, organ weights, morphometric indices of jejunum, and DON metabolism in broilers. To the best of our knowledge, the present study is the first study to follow the passage of DON through subsequent segments of the digestive tract, blood, bile and in the liver with special consideration of the occurrence of de-epoxy-DON.

In the present study, the level of dietary zearalenone content was not high enough to cause any adversely affects on performance of broiler chickens, based on previous studies of Bacon and Marks [28] and Swamy *et al*. [29]. Moreover, a toxicological synergism between deoxynivalenol and zearalenone has not been observed in broiler chickens [29]. Furthermore, DON concentrations in the control diets (<0.1 mg/kg) were too low to cause altered performance in broiler chickens [30]. Most importantly, the very potent trichothecenes T-2 and HT-2 toxins were close to the detection limit in all diets and thus not responsible for the effects observed [31]. This justifies the conclusion, that the effects observed in the present study are attributable to DON.

### 3.1. Birds Performance

All birds appeared clinically normal during the entire feeding trial. No mortality occurred over the course of the whole experiment. The general performance (BW; BWG and feed efficiency) of the birds are shown in Tables 2 and 3. The mean BW over the course of the experiment was not affected (*P* > 0.05) by DON contaminated diet at both levels (1 and 5 mg/kg). Additionally, the overall mean of BWG (1525 and 1562 g/bird) over the course of the whole experiment were not affected by the dietary inclusion of DON at both levels (1 and 5 mg/kg, *P* > 0.05) compared with the controls (1574 g/bird). The overall mean of feed intake (1836 and 1889 g/bird) were also not affected by the dietary inclusion of DON at both levels (1 and 5 mg/kg, *P* > 0.05) compared with the controls (1903 g/bird).

In fact, during the first week of the experiment, a significant decrease in feed intake in a quadratic (*P* = 0.012) way was observed for the chicks receiving the DON diet compared to the chicks fed the control diet. Additionally, in the second week of the experiment, a significant decrease in BW and BWG in a quadratic manner (*P* = 0.009, and *P* = 0.036, respectively) was observed for the chicks receiving the DON diet at both levels compared to the chicks fed the control diet. The results indicated that the adverse effects of DON contaminated diet on performance were mainly due to a depression in feed intake. However, this effect was especially pronounced at the beginning of the experiment. The results indicate that broilers of these groups were able to adapt to the contaminated diet over the course of the experiment. The BW and the efficiency of feed utilization over the course of the experiment were not adversely affected (*P* > 0.05) by DON in the diets.

The results obtained in the present study are indeed comparable with the results obtained previously after feeding DON contaminated diet of broiler chickens [11,29,32]. Dänicke *et al*. [9] reviewed the literature regarding the effects of DON on the performance of broilers and came to the conclusion that dietary concentrations greater than 5 ppm are necessary to cause detrimental effects. Even higher concentrations did not consistently induce detrimental effects and in fact, even growth promoting effects were observed especially at moderately high concentrations of DON. Furthermore, most experimental studies [29,32–37] with poultry show a highly variable effect of DON on performance indicating that zootechnical traits might not be a sensitive indicator of toxicity of this *Fusarium* toxin. However, feed refusal and reduced weight gain can be found when the dietary concentration of DON reached 16–20 ppm [38,39].

### 3.2. Organ Weights

The effects of feeding DON-contaminated diets at different levels (1 and 5 mg/kg) on the absolute and relative weights of the liver, heart, proventriculus, gizzard, small intestine, spleen, pancreas, colon, cecum, bursa of Fabricius and thymus at 35 days of age are shown in Tables 4 and 5. The absolute and relative weights of organs (liver, heart, proventriculus, gizzard, small intestine, spleen, pancreas, colon, cecum, bursa of Fabricius and thymus) remained unaltered by DON-intake (*P* > 0.05).

The effects of feeding DON-contaminated grains on organ weights of broiler chickens are very contradictory. Kubena *et al*. [40] found that the absolute and relative weights of the liver were decreased in growing chicks fed DON contaminated grains. Furthermore, Kubena and Harvey [41] observed no changes in organ weights (liver, spleen, kidney, and bursa of Fabricius). In another study, Kubena *et al*. [38] reported increased weight of bursa of Fabricius. In all these studies the chickens were fed 16 mg of DON/kg from contaminated wheat for 21 days. The outcome of these studies was highly variable, indicating that organ weights might not be a relevant indicator of toxicity of some *Fusarium* mycotoxins. The exposure time of the toxin may be a significant factor for toxin effects on organ weights because the organ initially swells with a short-time exposure followed by shrinkage with long-time exposure [42].

Additionally, no significant (*P* > 0.05) difference was noticed between birds fed DON and the control for the intestine length. For the intestine density (weight/length ratio), no significant difference was noticed between all treatments (Table 6). There was a positive correlation between small intestine weight and its length (*P* = 0.035, *r* = 0.452) and a positive correlation between the small intestine length and bird body weight (*P* = 0.029, *r* = 0.467).

### 3.3. Morphometric Indices

Following ingestion of contaminated feed, the intestinal epithelium can be exposed to high concentrations of DON [2]. Consequently, the digestive tract is a primary target to elicit toxic effects. In the present study, a significant reduction of VH, AVSA, and muscularis thickness in jejunum was observed after feeding contaminated grains. These histological alterations could be attributed to the irritant effects of DON on the upper gastrointestinal tract. These changes might appear as indirect response to the dietary trichothecenes. Moreover, DON inhibits protein synthesis and this causes necrosis of epithelial cells. So, the tissue most affected in the manner is the lining of the intestinal tract. This can cause bleeding into the intestinal lumen, increased frequency of ulcers and damage to the absorptive surfaces causing reduced nutrient uptake. In addition, Rocha *et al*. [43] reported multiple inhibitory effects for trichothecenes on eukaryotic cells including disruption of normal cell function by inhibiting RNA, DNA, and inhibition of cell divisions, stimulation of ribotoxic stress response, and activation of mitogen-activated protein kinases. The latter enzymes catalyze reactions in signal transduction related to proliferation, differentiation, and apoptosis [5].

**Figure 1:**
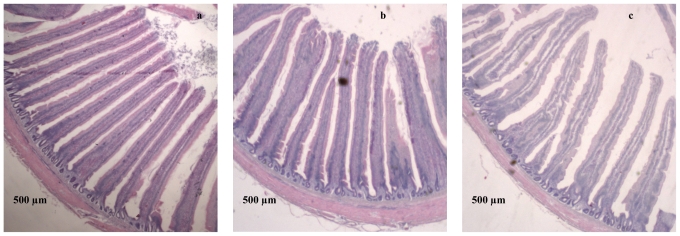
Histomorphometric analysis of the jejunum of a 5-wk-old broiler chickens fed diets with or without DON with magnification 200 (*n* = 6): (**a**) the Villus height of the jejunum of the control birds; (**b**) birds fed with 1 mg DON/kg diet; (**c**) birds fed with 5 mg DON/kg diet.

In the present study, deoxynivalenol at both levels significantly altered the small intestinal morphology (Table 7, and Figure 1). In the jejunum, the villi were shorter (*P* < 0.01) (1200 μm and 1288 μm for 1 mg and 5 mg DON/kg diet) in DON fed birds compared with the controls (1528 μm). The dietary DON contamination also decreased (*P* < 0.05) the villus surface area (188 mm^2^ and 203 mm^2^ for 1 mg and 5 mg DON/kg diet) in DON fed birds compared with the controls (315 mm^2^). However, no dietary effect was apparent for villus width, crypt depth, and villus height/crypt depth ratio in the jejunal mucosa. Furthermore, there was no correlation between the villus height and the small intestinal weight (*P* = 0.102, *r* = 0.398).

Polynomial contrasts constructed also on morphological parameters resulted in a significant effect of diets on villus length linearly (*P* = 0.036) and quadratically (*P* = 0.006). Additionally, dietary incorporation of contaminated grains resulted in a linear (*P* = 0.046) and quadratic response (*P* = 0.040) in villus area. The increasing levels of dietary DON were found to decrease the jejunal muscularis thickness in a linear (*P* = 0.034) way. However, there was no response of DON contaminated diets (*P* > 0.05) on the other morphometrical variables.

The results obtained in the present study are indeed comparable with the results obtained previously after feeding DON contaminated diet of broiler chickens [12]. Girgis *et al*. [44] found that feeding of diets contaminated with *Fusarium* mycotoxins could alter intestinal morphology in broiler breeder. Fairchild *et al*. [45] reported significant reduction in relative intestinal weight and jejunal serosa thickness in turkey poults fed 300 mg of purified fusaric acid (FA)/kg of feed for 18 days. Feeding 4 mg of diacetoxyscirpenol (DAS)/kg of feed to turkey poults did not affect the weight of intestine; however, feeding both, FA and DAS, to poults decreased enterocyte height at midvillus by 59%. This decrease, however, is indicative of *Fusarium* mycotoxins altering digestive and absorptive function [13,45]. This is supported by Sklan *et al*. [46] who indicated that feeding of T-2 toxin or diacetoxyscirpenol at levels up to 1 ppm for 32 days to poults did not depress but enhanced growth, and did not influence antibody production but caused changes in small intestinal morphology, especially in the jejunum where villi were shorter and thinner.

Trichothecenes cause harmful injury to the mucosa, destroying cells on the tips of villi and radiomimetic injury to rapidly dividing crypt epithelium [47]. The morphological alterations in villus height, muscularis thickness, and AVSA may contribute to reduced nutrient absorption in jejunum as we have previously reported [11,48]. However, in the present study, no significant difference was noticed between all treatments for the intestine density (weight/length), indicating that the intestine density might not be a sensitive indicator of toxicity of this *Fusarium* toxin.

### 3.4. Deoxynivalenol Tissue Distribution

Very little is known about the metabolism and kinetics of DON when it passes through the digestive tract, which is the first step governing the entry of DON into the organism. It is known from the literature that DON is metabolized by rumen microbes and microbes from large intestine of hens to de-epoxy-DON [18,49]. In the present study, the DON concentrations after oral exposure was rapidly and nearly completely absorbed (90%) while passing through the stomach and resulted in the distinctly faster disappearance of DON from this part of the digestive tract. Moreover, absorption of DON from the stomach could have contributed to the rapid DON disappearance. Such a possibility was discussed by Awad *et al*. [13], who detected DON in the blood side of broilers as early as 30 min after exposure to DON. Whether the 90% absorbed DON was systemically available cannot be answered since any simultaneous measurement of DON-kinetics after oral DON exposure was undertaken and thus the contribution of a possible hepatic and renal first-pass effect to DON-elimination remains to be answered. There is scarce information available about the role of bile and biliary excretion of DON in its overall metabolism in chickens.

In the current experiment, DON and DOM-1 were analyzed in serum and digesta from consecutive segments of the digestive tract (gizzard, cecum, and rectum). The DON concentration in gizzard was about 10 to 12% of DON intake irrespective to the dietary DON-concentration. Only about 6% of the ingested DON was recovered in feces and about 18 to 22% was recovered in cecum as unchanged DON irrespective to the dietary DON-concentration. Neither DON nor DOM-1 was detected in any of the analyzed specimens (blood, liver, and bile). However, there was a positive correlation (*P* = 0.000) between the amount of DON recovered in the digesta and the amount of the ingested DON. DON concentration in the digesta increased (*P* = 0.000) in a dose dependent manner (Table 8). In the present study, samples from small intestine digesta were not taken because of the low dry matter content of <10% and, in addition, many times the small intestine was empty.

The results indicate an effective elimination of the toxin from the broiler’s body, which is in contrast to pigs in which DON residues can be measured in blood and bile, even when the diets contained <1 mg of DON/kg of diet [50–52]. Additionally, only about 6% of the ingested DON was recovered in feces and about 20 % o was recovered in cecum as unchanged DON. The high amount of DON in the cecum could be attributed to the passage of DON is related to the dry matter content which might indicate that DON probably emptied with the liquid phase of the digesta. It might also be attributed to the urinary back reflux from cloacae and lead to a high absorption of DON. The results of the present study are in agreement with Lun *et al*. [53], who reported that DON seems to be rapidly and efficiently absorbed, most probably from the stomach and upper parts of the small intestine and little appears in the excreta of hens.

In the present trial, DOM-1 did not appear in the large intestine and in feces (Table 8). The results indicate that de-epoxydation in the present study hardly occurred in the distal segments of the digestive tract, assuming that the complete de-epoxydation occurs in the proximal small intestine where the majority of the parent toxin is absorbed. It seems reasonable to assume that the nearly complete de-epoxydation in the small intestine contributes highly to a detoxification of ingested DON. However, this was not investigated in the present study. This view is further substantiated by the results of He *et al*. [18] who reported that the intestinal microflora of chickens plays a major role in DON detoxification which might explain the relative tolerance of poultry.

In general, the recommended maximum level of DON in grains used in poultry diets is 5 mg/kg. The level of DON in the experimental diets of the current study is, therefore, of practical relevance to the field. The feeding of DON contaminated diets in the current study represents chronic exposure of broiler chickens to DON rather than an acute toxin challenge. Interestingly, no significant differences were found among the DON-supplemented groups for morphologic changes in the intestine (villus length and absorptive area), indicating that low and moderate concentrations of DON exhibit a similar toxicity. The findings reported here under experimental conditions assume practical importance because of the common occurrence of *Fusarium* mycotoxins in poultry feeds.

Moreover, understanding the mechanistic basis by which these toxins cause toxicity in chickens will improve our ability to predict the specific thresholds for adverse effects as well as the persistence and reversibility of these effects in the highly susceptible animals and human.

## 4. Conclusions

In conclusion, diets with DON contamination below levels that induce a negative impact on performance could affect the animal health by alteration of small intestinal morphology in broilers. Additionally, the results confirm that the majority of the ingested DON is quickly, nearly completely, absorbed in the stomach and proximal part of the small intestine. It was concluded that de-epoxydation of DON did not occur in the hindgut, suggesting that complete de-epoxydation occurs in the proximal small intestine, contributing highly to a detoxification of ingested DON in broilers.

## Figures and Tables

**Table 1:** Proximate and mycotoxin analysis of experimental diets 1.

Item	Group		

	Control	DON 2 (1 mg/kg)	DON 2 (5 mg/kg)
**Broiler starter**			
DM %	91.2	91.6	91.2
CP %	21.7	21.5	21.8
Crude fiber %	2.4	2.7	2.9
Crude fat %	7.7	7.8	7.7
Crude ash %	5.6	5.5	5.9
**Mycotoxins (μg/kg)**^3^			
Deoxynivalenol	42.7	822	5017
3-Acetyl-deoxynivalenol	4.5	17.2	114
Zearalenone	4.2	75.6	352
T-2 toxin	3.7	<1.50	<1.50
HT-2 toxin	<5.00	<5.00	<5.00
**Broiler grower**			
DM %	91.7	91.7	91.5
CP %	19.5	18.9	18.9
Crude fiber %	2.7	2.7	2.9
Crude fat %	9.7	9.0	9.8
Crude ash %	5.4	5.0	5.4
**Mycotoxins (μg/kg)**^3^			
Deoxynivalenol	87.6	872	4589
3-Acetyl-deoxynivalenol	5.0	18.7	100
Zearalenone	13.8	110	334
T-2 toxin	6.0	<1.50	<1.50
HT-2 toxin	7.0	6.9	<5.00

1 Proximate analysis on DM basis for nutrient content according to Naumann and Bassler (2004);

2 DON = deoxynivalenol;

3 Mycotoxin analysis by the LC-MS/MS method according to Błajet-Kosicka *et al*. (2008) and the limit of detection for deoxynivalenol, 3-Acetyl-deoxynivalenol, T-2 toxin, HT-2 toxin and zearalenone: 7.0, 5.0, 0.5, 1.7, and 0.1 ng/g, respectively.

**Table 2:** Body weights (g/bird) of the experimental birds (*n* = 15) 1.

Dietary treatment	Age 2				

	W1	W2	W3	W4	W5
**Control**	132	397	710	1271	1616
**DON**^3^**(1 mg/kg)**	116	292	642	1195	1567
**DON**^3^**(5 mg/kg)**	122	377	730	1220	1604
**Linear**	0.383	0.688	0.615	0.388	0.858
**Quadratic**	0.388	0.009	0.127	0.373	0.591
**PSEM**^4^	5	18	19	22	19

1 *n* = number of birds;

2 Values are the means of 5 pens; the pen with the group of 3 broilers was the experimental unit for performance data;

3 DON = deoxynivalenol;

4 PSEM = pooled SEM.

**Table 3:** Effects of dietary *Fusarium* mycotoxin deoxynivalenol on performance of male broilers (*n* = 15) 1.

Item	Live weight gain (g/bird) 2					Feed intake (g/bird) 2					Feed: gain (g/g) 2				

	W1	W2	W3	W4	W5	W1	W2	W3	W4	W5	W1	W2	W3	W4	W5
**Control**	90	265	355	561	345	105	274	456	671	549	1.181	1.055	1.319	1.195	1.594
**DON**^3^**(1 mg/kg)**	74	176	346	553	373	85	222	410	684	590	1.154	1.266	1.263	1.240	1.580
**DON**^3^**(5 mg/kg)**	80	256	354	490	384	102	276	466	628	606	1.328	1.080	1.312	1.283	1.582
**Linear**	0.401	0.845	0.997	0.033	0.066	0.786	0.964	0.844	0.254	0.122	0.363	0.855	0.981	0.081	0.817
**Quadratic**	0.420	0.036	0.988	0.066	0.172	0.012	0.098	0.661	0.308	0.291	0.526	0.176	0.914	0.231	0.957
**PSEM**^4^	5	17	26	14	9	4	12	26	16	15	0.062	0.050	0.053	0.020	0.020

1 *n* = number of birds;

2 Values are the means of 5 pens; the pen with the group of 3 broilers was the experimental unit for performance data;

3 DON = deoxynivalenol;

4 PSEM = pooled SEM.

**Table 4:** Effects of dietary *Fusarium* mycotoxin deoxynivalenol on absolute organs weights of broiler chickens (g, *n* = 7) 1.

Dietary treatment	Proventriculus	Gizzard	Small intestine	Liver	Cecum	Colon	Spleen	Bursa of Fabricius	Thymus	Pancreas	Heart
**Control**	7.7	40.2	54.9	30.1	13.3	4.2	1.5	2.9	5.7	2.6	8.4
**DON**^2^**(1 mg/kg)**	6.4	35.4	55.0	31.8	10.8	2.9	1.1	3.0	4.7	2.5	7.8
**DON**^2^**(5 mg/kg)**	6.9	42.2	57.4	32.9	13.3	4.6	1.6	2.9	4.8	2.8	7.9
**Linear**	0.462	0.622	0.659	0.514	0.996	0.755	0.624	0.926	0.197	0.607	0.426
**Quadratic**	0.396	0.190	0.882	0.810	0.422	0.297	0.137	0.982	0.282	0.729	0.539
**PSEM**^3^	0.4	1.6	2.2	1.7	0.9	0.5	0.1	0.2	0.3	0.1	0.2

1 *n* = number of birds;

2 DON = deoxynivalenol;

3 PSEM = pooled SEM.

**Table 5:** Effects of dietary *Fusarium* mycotoxin deoxynivalenol on organs weights relative to body weight of broiler chickens (%, *n* = 7) 1.

Dietary treatment	Proventriculus	Gizzard	Small intestine	Liver	Cecum	Colon	Spleen	Bursa of Fabricius	Thymus	Pancreas	Heart
**Control**	0.51	2.66	3.63	1.98	0.94	0.28	0.10	0.19	0.38	0.17	0.55
**DON**^2^**(1 mg/kg)**	0.43	2.37	3.68	2.10	0.71	0.19	0.07	0.20	0.31	0.17	0.52
**DON**^2^**(5 mg/kg)**	0.44	2.63	3.59	2.03	0.82	0.29	0.10	0.18	0.30	0.17	0.50
**Linear**	0.248	0.899	0.890	0.822	0.384	0.820	0.950	0.770	0.097	0.984	0.273
**Quadratic**	0.363	0.295	0.964	0.865	0.197	0.331	0.131	0.878	0.196	0.971	0.534
**PSEM**^3^	0.02	0.08	0.13	0.09	0.05	0.03	0.00	0.01	0.02	0.00	0.02

1 *n* = number of birds;

2 DON = deoxynivalenol;

3 PSEM = pooled SEM.

**Table 6:** Intestinal traits of broiler chickens fed diets with different levels of deoxynivalenol (*n* = 7) 1.

Dietary treatment	Parameters	

	Intestine length, cm	Intestine density, weight/length (g/cm)
**Control**	131	0.43
**DON**^2^**(1 mg/kg)**	131	0.40
**DON**^2^**(5 mg/kg)**	125	0.44
**Linear**	0.557	0.808
**Quadratic**	0.800	0.765
**PSEM**^3^	3.68	0.02

1 *n* = number of birds;

2 DON = deoxynivalenol;

3 PSEM = pooled SEM.

**Table 7:** Effects of feeding *Fusarium* mycotoxin deoxynivalenol on small intestinal morphology of broiler chickens at 5 wk of age (*n* = 6) 1.

Dietary treatment	Parameters					

	Villus height (μm)	Crypt depth (μm)	Villus width (μm)	Muscularis thickness (μm)	Villus apparent surface area (mm^2^)	Villus-to-crypt ratio
**Control**	1528 a	136	227	238 a	315 a	11.40
**DON**^2^**(1 mg/kg)**	1200 b	118	179	170 b	188 b	10.21
**DON**^2^**(5 mg/kg)**	1288 b	133	196	160 b	203 b	9.83
**Linear**	0.036	0.718	0.466	0.034	0.046	0.125
**Quadratic**	0.006	0.114	0.507	0.070	0.040	0.287
**PSEM**^3^	48	4	16	16	23	0.41

1 *n* = number of birds;

2 DON = deoxynivalenol;

3 PSEM = pooled SEM;

a,b Means in column with a common superscript are significantly different (*P* < 0.05).

**Table 8:** DON 1 concentration in the content of gizzard, cecum, rectum and feces (*n* = 7) 2.

Dietary treatment	DON concentration (μg/kg)			

	Gizzard	Cecum	Rectum	Feces
**Control**	10.4 a	13.3 a	0.0 a	3.3 a
**DON**^3^**(1 mg/kg)**	109.9 b	173.7 b	34.0 b	51.3 b
**DON**^3^**(5 mg/kg)**	557.7 c	867.0 c	245.7 c	282.7 c
**Linear**	0.000	0.000	0.000	0.000
**Quadratic**	0.000	0.000	0.000	0.000
**PSEM**^4^	61.4	131.0	31.0	30.9

1 Concentrations of DON in gizzard, cecum, rectum and feces which were lower than the above indicated detection limits were considered with a concentration of zero in evaluating the data which implies that calculated mean values might be lower than the detection limits;

2 *n* = number of birds;

3 DON = deoxynivalenol;

4 PSEM = pooled SEM;

a,b,c Means in column with a common superscript are significantly different (*P* < 0.001).
